# Imitation, Rivalry, and Escalation: Rethinking Adolescent Self-Harm Through Mimetic Theory

**DOI:** 10.1007/s11013-025-09926-3

**Published:** 2025-07-05

**Authors:** Andrew Sweetmore

**Affiliations:** University of Bournemouth, Bournemouth, UK

**Keywords:** Self-harm, Adolescents, Social Media, Interventions, Theory

## Abstract

Self-harm amongst young people has risen significantly in recent years, yet existing models fail to fully explain its underlying mechanisms. This paper applies René Girard’s theory of mimetic rivalry and escalation to self-harm, proposing that competition for social status and identity within peer groups and families may contribute to its development. In this framework, self-harm operates as a form of self-punishment, mirroring Girard’s concept of scapegoating; a ritualised resolution to the tensions produced by mimetic escalation. The study explores how social media amplifies these dynamics by intensifying social comparison and reinforcing cycles of imitation and rivalry. Current treatments may be limited in their efficacy as they primarily focus on precipitating factors and crisis resolution without addressing the mimetic mechanisms driving self-harm. Integrating mimetic theory into clinical practice could offer a new framework for intervention, helping young people recognise and disengage from destructive social dynamics. Additionally, the paper highlights the potential for systemic and group-based interventions that target mimetic escalation within peer and family relationships. By understanding self-harm as a product of mimetic processes, this perspective offers novel insights for research, clinical practice, and public health strategies aimed at addressing the rise in self-harm amongst adolescents.

## Introduction

The prevalence of self-harm, defined as the intentional act of self-injury or self-poisoning, has increased significantly amongst young people over the last decade (Borschmann et al., 2017). The efficacy of current interventions is limited at best; treatments appear to benefit certain individuals under specific conditions, yet there remains no reliable way to predict for whom, when, or why they will be effective (Fox et al., 2020; Harris et al., 2022; Iyengar et al., 2018). Consequently, clinicians, policymakers, and researchers continue to call for a deeper understanding of the factors driving self-harm and the mechanisms underpinning its occurrence (Witt et al., 2021).

Despite over fifty years of research yielding numerous theories and treatments, a comprehensive understanding of self-harm remains elusive (Fox et al., 2020; Harris et al., 2022). Existing models of self-harm propose explanations such as self-punishment, which frames the behaviour as a means to alleviate guilt or shame (Nock, 2009); anti-dissociation, which views it as a way to counter emotional numbness (Chapman et al., 2006); care-seeking, which interprets its function as a signal for support (Klonsky, 2007); and contagion, which highlights its spread through social influence (Jarvi et al., 2013). These models often rely on predisposing factors, symptoms, and post hoc rationalisations to explain the behaviour (Iyengar et al., 2018; Kothgassner et al., 2020). These frameworks, however useful, fall short in clarifying what drives young people to self-harm (Prinstein et al., 2010) and underexplore the role of group dynamics, particularly within peer relationships and digital environments.

A similar gap exists in understanding the role of social media in the rise of self-harm. Whilst exposure to harmful content and online bullying are frequently cited as contributing factors for the worsening mental health amongst young people (Lee et al., 2022), these explanations remain incomplete. They do not explain why some individuals are more susceptible to social media’s assumed influence than others, or how it may influence a young person to self-harm. 

This paper proposes that an adaptation of René Girard’s theory of mimetic rivalry offers a useful framework for understanding self-harm in specific groups of young people. It also provides a possible theory into how and why self-harm emerges within adolescent social structures, particularly in relation to status competition and identity formation, and why mimetic escalation occurs in the first place. Furthermore, this perspective may explain why existing interventions appear to have only limited efficacy in reducing self-harm amongst young people.

## Mimetic Theory and Its Application to Self-Harm

René Girard, an anthropologist and literary critic, developed the theory of mimetic desire, which suggested that human desires are not arrived at autonomously, but are shaped through imitation of others (Girard, 2023, 2024). Girard stated that we desire objects or goals not spontaneously but by emulating a "model," creating a triangular structure of subject (the desirer), model (the one imitated), and object (the desired entity or status) (Girard, 2024). This mimetic process fosters social cohesion by aligning desires within a group (Girard, 2024), however, imitation, fundamental to social learning, can obscure individuality when people, especially adolescents seeking identity and belonging in competitive settings like peer groups or families, conform too closely (Allen et al., 2020; Meehan et al., 2024). This loss of difference escalates into mimetic rivalry, where similarity, not difference, fuels conflict over shared objects or status, a process which Girard described as ‘undifferentiation’ (Girard, 2024). A typical example is a child who, upon seeing a peer play with a toy, desires that specific toy, not for its inherent appeal, but because it is valued by another, despite the availability of other toys.

Historically, societies curbed such tensions through scapegoating, redirecting collective aggression towards an individual or group to restore harmony (Girard, 2024). Girard cautioned, however, that this ritualised violence depended on its concealment; if recognised as unjust, the scapegoat’s victimhood would undermine its efficacy (Girard, 2001, 2024). To contain this rivalry, he emphasised the necessity of strong social prohibitions against coveting others’ possessions or status, lest undifferentiation intensify conflict (Girard, 2023).

Modern societies, however, are characterised by a relative absence of such prohibitions, particularly concerning the pursuit of status and material possessions (Zizek, 2009). Whilst legal frameworks exist, social and cultural restrictions on ambition, consumption, and competition are minimal (Schmitt et al., 2022). What remains strongly prohibited is direct violence, towards both others and oneself. Self-harm, therefore, becomes significant in cultures where outward violence is restricted but internalised violence remains unregulated in all but extreme cases.

## A Model of Self-Harm as Mimetic Escalation

Adolescents are highly susceptible to peer influence, shaping their behaviours, choices, and social identities through conformity to group norms (Allen et al., 2020; Bosacki et al., 2007; Laursen & Veenstra, 2021; Meehan et al., 2024). The search for individual identity marks a key developmental milestone in adolescence (Acheson & Papadima, 2023). From a Girardian perspective, this process reflects mimetic desire, as young people model their aspirations on those perceived as socially desirable (Allen et al., 2020; Girard, 2024). Social comparison, an inevitable facet of identity development, is central to Girard’s concept of individuality, which posits that identity forms relationally through interactions with others (Girard, 2024).

Uh et al. (2021) outline two primary pathways to self-harm: a psychopathology pathway and an adolescent risky behaviour pathway. The framework proposed here aligns with both, though its applicability may be limited for individuals with severe psychopathology, such as chronic depression, psychosis, or suicidality. Whilst contagion models explain the spread of self-harm behaviours within peer networks (Jarvi et al., 2013; Swanson & Colman, 2013), Girardian theory offers a deeper perspective, revealing how mimetic desire escalates beyond passive transmission into rivalry, social tension, and conflict (Girard, 2024). Crucially, it should be recognised that mimetic rivalry and escalation are largely unconscious processes, which makes them difficult to recognise and address directly. 

Self-harm within the proposed mimetic model typically occurs in one of two broad pathways:

1. Mimetic escalation within peer groups
2. Mimetic escalation within families

Within peer groups, further subcategories emerge:

### Peer Group A: Self-Harm as a Ritualised Practice

The young person belongs to a group where self-harm is an established behaviour, acting as a form of meaning-making. Here, the model and object of desire may be a highly regarded peer or a public figure (e.g., a musician) who the group seeks to emulate. Violence towards the self is not prohibited within this context; instead, it is transgressive, violating societal taboos whilst simultaneously enhancing status within the group. Self-harm may be documented, shared, and reinforced as a positive act through digital platforms, further entrenching the behaviour within a mimetic cycle. Here, self-harm is an inclusionary practice within the group.

### Peer Group B: Self-Harm as a Consequence of Status Rivalry

In this group, self-harm is not part of the social identity, at least not initially. Instead, status is determined by desirable attributes such as appearance, academic achievement, athletic performance, social media presence, or wealth. Young people observe which peers attain the highest status and attempt to emulate their traits. However, status competition is inherently exclusionary, not all members can achieve the same level of success or ability. Within this group we primarily see the mechanism of undifferentiation and its consequences.

Within these two groups mimetic rivalry can manifest in two ways:

- The young person competes for status, leading to escalation of behaviour, and possibly exhaustion, frustration, distress, and eventual crisis. The young person may not view this escalation (increase in self-harm) as a problem requiring a clinical solution but is likely an issue for caregivers and any professionals involved in the young persons life.
- The young person competes for status or simply belonging as a member of the group. Due to mimetic desire their presence causes scandal and disrupts the group’s hierarchy, resulting in exclusion or bullying as a form of scapegoating, utilised in order to maintain social equilibrium.

### Mimetic Escalation Within Families

Within families, the object of desire is parental affection, with a parent or sibling serving as the model. Mimetic escalation occurs in three primary ways:

- **Sibling Rivalry:** Competition for parental attention, often expressed through achievement, rule-breaking, or health-related concerns. Attention may be a substitute for love in the short term. The young person may have learnt that when they are unwell they receive care and sympathy from their parent, which can act as a shortcut to affection.
- **Rivalry with the Model Parent:** One parent serves as the object of desire, whilst the other acts as the model. The young person seeks to supplant the model parent by monopolising the attention of the object parent, utilising the behaviours previously mentioned.
- **Rivalry with the Object Parent:** The same patterns of behaviour emerge but within an invalidating environment where the object caregiver is absent, physically or emotionally. In some cases, the young person may become an obstacle to the object parent's own desires, creating a mimetic rivalry between parent and child, and open conflict.

This family dynamic accords with Fairbairn’s (1952) observation that children may internalise blame for parental dysfunction, preserving emotional attachment and family cohesion by adopting a scapegoat role. Within families, where parental affection is the desired object and a caregiver the model, a young person’s mimetic rivalry, seeking love through health-related concerns, may thus intensify self-blame and conflict.

Regardless of the pathway, mimetic escalation inevitably leads to a crisis. When external violence is prohibited, self-harm becomes a means of resolving the conflict.

## Mimetic Rivalry and Self-Harm

Girard states that mimetic rivalry is a pervasive force in all human cultures, particularly modern capitalist societies (Girard, 2024), yet it does not always lead to self-harm. Therefore, what differentiates those who engage in self-harm from those who do not?

In each of the pathways described, mimetic escalation generates conflict. The pressures to succeed, to conform to idealised standards, to seek affection but not receive it, or to remain constantly engaged in digital communication can be psychologically demanding. It is unsurprising, then, that self-harm has increased alongside rising levels of mental health difficulties amongst young people (Kothgassner et al., 2020).

Within this mimetic cycle, shame and self-blame may emerge. A young person who perceives themselves as failing to imitate the model, whether in appearance, social status, or perceived success, may experience a profound sense of inadequacy. If this failure is accompanied by bullying or social exclusion, the tension escalates further. However, one principle remains constant: direct violence towards others is socially prohibited, particularly directed at members of a social group. Instead, aggression is internalised. Self-harm, perceived as a means of catharsis, becomes a ritualised practice of self-punishment, self-victimisation, or, in Girardian terms, *self-scapegoating* (Girard, 2024). Within certain peer groups (*Peer Group A, above*), self-harm and subsequent self-victimisation may even acquire symbolic meaning, reinforcing identity and group cohesion (Jarvi et al., 2013).

Girard (1996) observed that eating disorders, particularly anorexia nervosa, manifest as mimetic struggles where individuals compete for social status through extreme self-denial. Strand (2018), in a formulation of Girard’s paper (1996), describes Girard’s conception of anorexia as an “inverted mimesis”, a “mimesis of renunciation”- a paradox echoed in self-harm’s symbolic nature, where causing pain serves to alleviate pain or induce positive emotional states, underscoring the ritualistic significance some individuals may attach to such acts. This similarity supports the idea that self-harm, like disordered eating, may arise from cycles of thwarted differentiation, particularly when other typical avenues for social recognition, such as normative conceptions of appearance or achievement, are no longer viable, or viewed as desirable by the peer group.

Girard’s theory of scapegoating suggests that when mimetic rivalries escalate to a crisis point, social cohesion is maintained by projecting hostility onto a single victim (Girard, 2024). This process temporarily restores equilibrium within the group. Some individuals, particularly those experiencing heightened guilt or social isolation, may unconsciously assume the role of the scapegoat (Girard, 2023). Fairbairn (1952) observed that within families, a child may internalise blame, acting in a way to maintain environmental stability or avoid conflict, preferring to see themselves as ‘bad’ rather than acknowledge unloving caregivers. Such dynamics, marked by escalating conflict, are associated with a heightened risk of self-injury (Yates, 2004). Though seemingly voluntary, this behaviour often represents a learned survival strategy within dysfunctional family systems (Fairbairn, 1952).

Research suggests that self-harm can spread within peer networks, exhibiting contagion effects that amplify its prevalence (Pitman et al., 2023; Prinstein et al., 2010). Studies indicate that the presence of a single individual who engages in self-harm within a group where such behaviour was previously absent can trigger an increase in self-harming behaviours amongst other members (Pitman et al., 2023), reinforcing the role of social influence in its transmission.

Prinstein et al. (2010) propose that adolescent socialisation may exert pressure on individuals to imitate the behaviours of their peers or adhere to social norms, increasing their susceptibility to peer influences on self-harm. However, whilst this explanation acknowledges the role of peer influence, it does not fully account for the underlying mechanism through which this process occurs. Similarly, recent studies highlight the feelings of guilt, shame and inferiority as precipitating factors for self-harm in young people (Mahtani et al., 2019; Xavier et al., 2016). Additionally, negative self-perception, self-criticism, and struggles with self-compassion further heighten vulnerability to self-harming behaviours (Gilbert et al., 2010; Selby et al., 2013). Whilst these studies explain what may influence these feelings to emerge, such as bullying, childhood maltreatment, invalidating environments (Liu et al., 2018; Mahtani et al., 2019; Muris & Meesters, 2014), they do not explain what gives rise to those acts occurring in and of themselves, and why this then leads to self-harm. Mimetic theory provides such a framework, explaining how the imitation of desires and behaviours, particularly within competitive or identity-forming peer and family dynamics, may drive the escalation of self-harm within these groups.

Whether a young person engages in self-harm may be influenced by predisposing risk factors (Knipe et al., 2022; McEvoy et al., 2023) alongside the extent to which they experience mimetic escalation beyond their ability to control or tolerate. However, as with other areas of psychiatry (Uher, 2014), disentangling the interplay between genetic predisposition and environmental influences for self-harm remains complex. If a young person internalises feelings of inferiority, shame, or guilt resulting from mimetic escalation, self-harm may serve as a means of resolving this distress. Conversely, those who become aware of the mimetic cycle may be able to disrupt its progression before it reaches crisis, preventing the adoption of self-harming behaviours as a ritualised response.

## Social Media as an Accelerator of Mimetic Escalation

Whilst self-harm has always existed in one form or another (Chaney, 2019), its prevalence amongst young people has increased markedly in the past decade (Kothgassner et al., 2020). The question, then, is what has changed?

This paper argues that social media accelerates mimetic desire and rivalry by amplifying their effects. The issue is not merely the content young people consume or the cognitive effects of excessive screen time (Vanderloo et al., 2020). Rather, the immediacy that digital technology facilitates intensifies the mimetic process itself. This mechanism provides an explanation for the disparities in how social media affects different individuals—why some are particularly susceptible whilst others remain largely unaffected. Furthermore, the belief that simply restricting young people’s access to social media will eliminate self-harm is likely misguided, as it fails to address the underlying social dynamics and psychological mechanisms, such as mimetic rivalry and social comparison, that drive the behaviour.

Burgis (2021) observes that the erosion of clear hierarchies in modern society, exacerbated by social media, fosters a ‘hyper-mimetic’ environment where young people rapidly cycle through unstable models of desire, heightening identity uncertainty, amplifying imitative impulses, and increasing psychological stress. The constant visibility of others spurs comparison and intensifies fear of missing out (FOMO). Digital technologies facilitate uninterrupted peer interaction, further fuelling these dynamics. Continuous exposure to idealised peers on social media may heighten feelings of inadequacy and exclusion (McComb et al. 2023), which, combined with mimetic rivalry, may drive some adolescents to self-harm as a way of managing emotional distress. This pattern extends to familial mimetic rivalry, where a young person, already in conflict with a carer or sibling, encounters idealised media portrayals of adult-child bonds, deepening feelings of exclusion. In this scenario, the family cohort exemplifies what Uh et al. (2021) describe as a *psychopathological pathway*.

Goodyear et al. (2025) report that smartphone bans in schools do not enhance students’ mental health or academic performance, despite a correlation between excessive phone use and poorer mental health outcomes. This finding supports a mimetic framework, as restricting digital platforms fails to mitigate rivalry within peer networks. However, Beland and Murphy (2016) demonstrate improved academic performance following smartphone bans, whilst Haidt (2025) highlights methodological flaws in studies finding no effect, underscoring an unresolved debate on digital restrictions and wellbeing. Addressing mimetic rivalry may thus be essential to alleviate psychological distress, beyond merely limiting technology.

The relative absence of social media-driven mimetic escalation leading to self-harm in adults may, paradoxically, support the argument that mimetic rivalry is central to its amplification. This difference could be attributed to adolescent brain development, making them more reactive to social influences compared to adults (Welborn et al., 2016). Additionally, the heightened importance of peer group belonging, the search for identity, and the greater availability of time to engage in mimetic escalation on a large scale may further contribute to this phenomenon.

## Why Current Treatments May Have Limited Efficacy

Social media can intensify mimetic rivalry, yet current self-harm interventions, which often focus on individual factors, may not sufficiently address the wider social dynamics driving this behaviour. Standard treatments, typically operating within a cognitive-behavioural framework, target maladaptive thoughts, emotional regulation, and distress tolerance to resolve immediate crises (Iyengar et al., 2018; Kothgassner et al., 2020). However, by neglecting relational dynamics like mimetic escalation, these interventions may only partially alleviate distress, leaving the social forces sustaining self-harm unaddressed.

Some treatment modalities may inadvertently engage with aspects of mimetic rivalry and escalation through their focus on interpersonal relationships. However, these mimetic processes are rarely acknowledged explicitly. For example, therapeutic interventions frequently encourage young people to develop coping strategies or therapeutic distraction techniques (Witt et al., 2024). Whilst these approaches can be beneficial, they do not necessarily disrupt the underlying mimetic cycle. From a Girardian perspective, what is a skill if not another form of ritualised behaviour? Skills are learned through teaching and imitation, often modelled by therapists or caregivers, and can themselves become part of a self-reinforcing cycle of behaviours aimed at maintaining social equilibrium. If interventions fail to recognise that social comparison and imitation are one of the key drivers of distress, they risk reinforcing rather than dismantling the mechanisms that sustain self-harm.

Furthermore, current frameworks tend to conceptualise self-harm retrospectively, analysing the factors leading up to and perpetuating an episode but often overlooking the underlying mechanism of how it occurs and why self-harm is chosen (Pitman et al., 2023; Yan & Yue, 2023), particularly within digital and social spheres. Many intervention strategies focus on risk assessment, crisis management, and harm reduction (Yan & Yue, 2023)—necessary but insufficient approaches if the behaviour is driven by mimetic escalation but not taken into account. For example, whilst safety plans and emotional regulation techniques may help manage acute distress (Witt et al., 2021), they do not necessarily address the broader relational dynamics that shape and reinforce self-harming behaviours, particularly when they are amplified by digital technology. If self-harm functions as a resolution to the tensions and a crisis produced by mimetic rivalry, then reducing individual distress without addressing the social conditions that generate it may have limited long-term impact.

This gap in our understanding on the mechanisms that drive self-harm is particularly evident in the role of social media, where cycles of imitation and competition are intensified. Existing digital well-being initiatives often focus on restricting content or reducing screen time (Vanderloo et al., 2020) but do not engage with the core issue: how digital environments amplify mimetic rivalry. Social media platforms create an ecosystem where status, identity, and validation are continuously negotiated through visibility and engagement metrics. If young people engage in self-harm within a mimetic framework, then interventions must consider not only the content they consume but also the ways in which digital interactions sustain patterns of rivalry and self-comparison.

## Implications for Clinical Practice

If we accept mimetic escalation as a plausible hypothesis, *at least until it can be empirically tested*, then developing more effective interventions requires a shift in focus: from treating self-harm as an isolated behavioural issue to understanding it within the wider context of social dynamics and mimetic desire as a consequence of neurobiological process. Mahtani et al. (2019) highlight the importance of addressing peer relationships as a driver of self-harm, but a Girardian-informed approach extends this by helping young people recognise and disengage from harmful mimetic cycles that underpin these interactions. This perspective offers a form of early intervention aimed at the root of psychological distress, intervening before self-harm occurs. Psychoeducational interventions could explore how social comparison shapes desires and behaviours, whilst therapeutic approaches might encourage critical reflection on rivalry and status competition in the development of distress. Within families, recognising patterns of mimetic escalation could form the basis for interventions that restructure relational dynamics, particularly in cases where parent-child or sibling rivalries contribute to emotional strain.

Applying the concept of mimetic escalation in clinical practice requires recognising the role it may play in the young person's presentation. Clinicians would need to assess whether mimetic rivalry is contributing to distress and whether treatment approaches are mitigating or inadvertently reinforcing it. For young people in groups where self-harm is ritualised, group therapy could help them recognise and disrupt mimetic cycles, whilst systemic therapy might address familial scapegoating dynamics that intensify self-blame. Girard stated that mimetic rivalry loses its power only when it is exposed (Girard, 2024), in therapeutic settings, this might involve working with young people to map their mimetic escalations, bringing the underlying mechanism into conscious awareness. This approach could complement existing cognitive-based interventions by enabling young people to identify and disengage from self-destructive mimetic cycles.

Systemic therapy, which focuses on relational dynamics within families or groups, may also provide a useful starting point for addressing the mimetic cycle of self-harm. However, existing systemic models do not explicitly account for mimetic rivalry in the strict Girardian fashion, nor are they typically applied to youth peer groups, where these dynamics may be most pronounced. An early intervention group therapy approach designed specifically for young people might be beneficial, offering a space to explore mimetic escalation within friendships and social circles. By making these unconscious processes visible, such interventions could help young people recognise the ways in which competition, status-seeking, and imitation contribute to psychological distress, and subsequently self-harm. Given the social nature of mimetic desire and its amplification through social spheres including online, school-based interventions may also provide a valuable setting for early identification and prevention.

Ultimately, if it is the case that for some self-harm is a response to mimetic escalation, then intervention must go beyond crisis resolution to focus on preventing escalation itself. This requires not only clinical adaptations but also broader cultural and policy shifts that acknowledge and address the ways in which individual biology, social structures and digital environments shape adolescent behaviour. Without such an approach, current interventions risk remaining reactive rather than transformative.

## Conclusion

Girard’s theory posits that mimetic rivalry is an inherent aspect of human behaviour, which has shaped social structures throughout history (Girard, 2023). Whilst eradicating rivalry between humans is likely impossible, recognising its role in self-harm offers a foundation for more effective interventions—ones that address not only the behaviour itself but also the mimetic forces that sustain it. By foregrounding the social mechanisms of mimetic rivalry, this perspective addresses a critical gap in existing models, which often overlook the relational dynamics sustaining self-harm. Understanding these dynamics is essential for developing treatment approaches that move beyond symptom management to address the deeper social mechanisms driving self-harm.
